# Tuberculosis and HIV/AIDS coinfection in patients attending Directly Observed Treatment Short‐course (DOTS) centers in Anambra State, Nigeria: A retrospective study

**DOI:** 10.1002/hsr2.2201

**Published:** 2024-06-17

**Authors:** Monique Okeke, Peter M. Eze, Adaeze E. Chukwudebelu, Chidiebere J. Nwankwo, Nchekwube K. Eze, Uchenna U. Okafor, Isaiah C. Abonyi, Eric E. Okereke, Kalu O. Obasi, Okorie A. Ede, Chika P. Ejikeugwu, Cajetan I. Ilo, Jerome O. Okafor

**Affiliations:** ^1^ Department of Environmental Health Science Nnamdi Azikiwe University Awka Anambra Nigeria; ^2^ School of Biological Science Queen's University Belfast Belfast UK; ^3^ Department of Pharmaceutical Microbiology and Biotechnology Nnamdi Azikiwe University Awka Anambra Nigeria; ^4^ Department of Pharmaceutical Microbiology and Biotechnology Enugu State University of Science and Technology Awka Anambra Nigeria; ^5^ Helmholtz‐Zentrum für Umweltforschung (UFZ) Leipzig Germany; ^6^ Department of Human Kinetics and Health Education Ebonyi State University Abakaliki Ebonyi Nigeria; ^7^ Department of Human Kinetics and Health Education Nnamdi Azikiwe University Awka Anambra Nigeria

**Keywords:** AIDS, Anambra, co‐infection, HIV, Nigeria, prevalence, TB, tuberculosis

## Abstract

**Background and Aim(S):**

This study retrospectively assessed the prevalence of TB and human immunodeficiency virus (HIV)/AIDS coinfection among patients that attended the Directly Observed Treatment Short‐course (DOTS) centers in Anambra State, Southeast, Nigeria, between 2013 and 2017.

**Methods:**

The study adopted a descriptive and retrospective epidemiological survey design. A total of 1443 case files of patients aged 15−60 who were treated in DOTS centers selected from Anambra State's 21 Local Government Areas between 2013 and 2017 were investigated. The uniform data form, a standardized instrument used in Anambra State's health facilities for data collection, was used to collect data from case files of all those identified as coinfected with TB and HIV/AIDS.

**Results:**

The mean prevalence rate of TB and HIV/AIDS coinfection in the state during the 5‐year period (2013–2017) was 20.00%. The highest annual prevalence of TB and HIV/AIDS coinfection was recorded in 2014 (23.84%). The state's prevalence of TB and HIV/AIDS coinfection increased dramatically from 13.17% in 2013 to 23.84% in 2014, followed by a slight downward trend to 22.80% in 2015, 20.17% in 2016, and 20.03% in 2017. In terms of gender, age, marital status, and occupation, females (59.5%), those aged 15 to 25 years (30.7%), married people (43.90%), and traders/business owners (50.7%), respectively, had the highest rates of tuberculosis and HIV/AIDS coinfection during the study period.

**Conclusion:**

The findings of this study show that young people, females, married people, and traders/business owners appear to be the most vulnerable groups affected by TB and HIV/AIDS coinfection, accounting for the majority of the disease burden in the state. To address the high prevalence of TB and HIV/AIDS coinfection in the Anambra State, novel intervention and control programs should be developed and implemented, and existing intervention frameworks should be strengthened.

## 1 INTRODUCTION

Tuberculosis (TB) is caused by the bacteria *Mycobacterium tuberculosis*, which typically affects the lungs, and is spread through the air, especially when people with pulmonary TB cough, sneeze, or spit.^1^ TB primarily affects people with reduced immunity, such as young children or people living with the human immunodeficiency virus (HIV), as well as those suffering from malnutrition, diabetes, or silicosis, and those who smoke or have substance use disorders. TB also disproportionately affects people whose health is compromised due to socioeconomic factors such as poverty, poor housing, displacement, or incarceration.^2^

People living with HIV are at increased risk of dying from TB, especially if TB is not diagnosed or is diagnosed late. HIV and TB form a lethal combination, each accelerating the progress of the other. In 2021, approximately 187,000 people died of HIV‐associated TB. High‐quality TB screening is critical to ensuring that people living with HIV receive timely treatment for TB disease or TB infection.^1^, ^3^

The WHO African Region has the highest burden of HIV‐associated TB, and countries in Sub‐Saharan Africa are the worst affected by the twin epidemic of TB and HIV. The prevalence of TB in the region, which hitherto was reported to be declining prior HIV epidemic, is now on the rise, with Nigeria among the countries with a high burden of TB.^1^, ^4^

TB remains a serious public health challenge in Nigeria, and the country ranks among the nations with the highest disease burden. TB has a negative impact on the country's growth and development because it causes both direct and indirect economic losses due to increased morbidity and mortality, and because a significant proportion of those affected are in productive age groups.^5^

Several TB control programs have been initiated in developing countries, most notably Nigeria, which has a high TB burden. They include the WHO‐instituted directly observed treatment short course (DOTS) program, DOTSplus, and Stop TB, all with the mandate of executing strategies aimed at reducing the global TB burden.^5^, ^6^, ^7^

Anambra State in Nigeria is a priority setting for TB control because it contributes significantly to the country's high TB burden, accounting for the highest prevalence in the South‐East region.^8^ Adebayo et al.^5^ conducted a health facility record for the year 2016, which included 22 DOTS facilities and 1281 TB treatment enrollees. They reported a prevalence rate of HIV/TB co‐infection of 24.4%, compared to 7.1% in Oyo State. Their findings revealed that TB treatment success and cure rates in Anambra State fell short of the WHO's recommended target of 85%.

Although a few other studies have reported on HIV and TB co‐infection in selected Anambra State health facilities and communities,^9^, ^10^, ^11^ there appears to be a scarcity of data on the prevalence of TB and HIV/AIDS co‐infection in most Anambra State communities. This has significant implications for effective planning, efficient resource allocation, and decisions about where and when to expand prevention and control activities in the state. The purpose of this study was to determine the prevalence of TB and HIV/AIDS co‐infection in patients who attended DOTS centers in Anambra State, Nigeria from 2013 to 2017.

## 2 METHODS

### 2.1 Research design

This study was a retrospective epidemiological survey of patients' clinical records from 2013 to 2017 to determine the prevalence of TB and HIV co‐infection in Anambra State, Nigeria, during that period. This retrospective study relied on secondary data, which was limited to the available and accessible information documented for patients who attended DOTS centers selected from each of the 21 Local Government Areas (LGA) in Anambra State between 2013 and 2017.

### 2.2 Study area and study population

This study was carried out in Anambra State, which is in Nigeria's South‐East geopolitical zone. According to the 2006 National Population Census, Anambra State has a population of over 4,055,048 people, distributed across the state's 21 LGA.^12^ Data from medical records of patients aged 15−60 who received treatments for TB and HIV/AIDS co‐infection at a DOTS center selected from each of Anambra State's 21 LGA between 2013 and 2017 were collected and analyzed. A total of 1443 accessible patients' case files/folders were sampled.

### 2.3 Data collection

The data used in this study was collected from the “uniform data form,” a standardized data collection tool designed specifically for collecting TB and HIV data from patients visiting Anambra State's DOTS centers. To ensure the integrity and authenticity of the data, no modifications or changes were made to the uniform data form. Data collected included the name of the DOTS facility providing the services, the year of the patient's treatment, the patient's age, gender, marital status, occupation, and other relevant information.

### 2.4 Data analysis

Data analysis was performed using Microsoft Excel (version 16.75.2). The annual prevalence of TB and HIV/AIDS co‐infection in Anambra State's 21 LGA from 2013 to 2017, and from 2014 to 2017, was compared using one‐way analysis of variance with a 95% confidence level. Indicator of statistical significance is *p* ≤ 0.05.

## 3 RESULTS AND DISCUSSION

**Figure 1:**
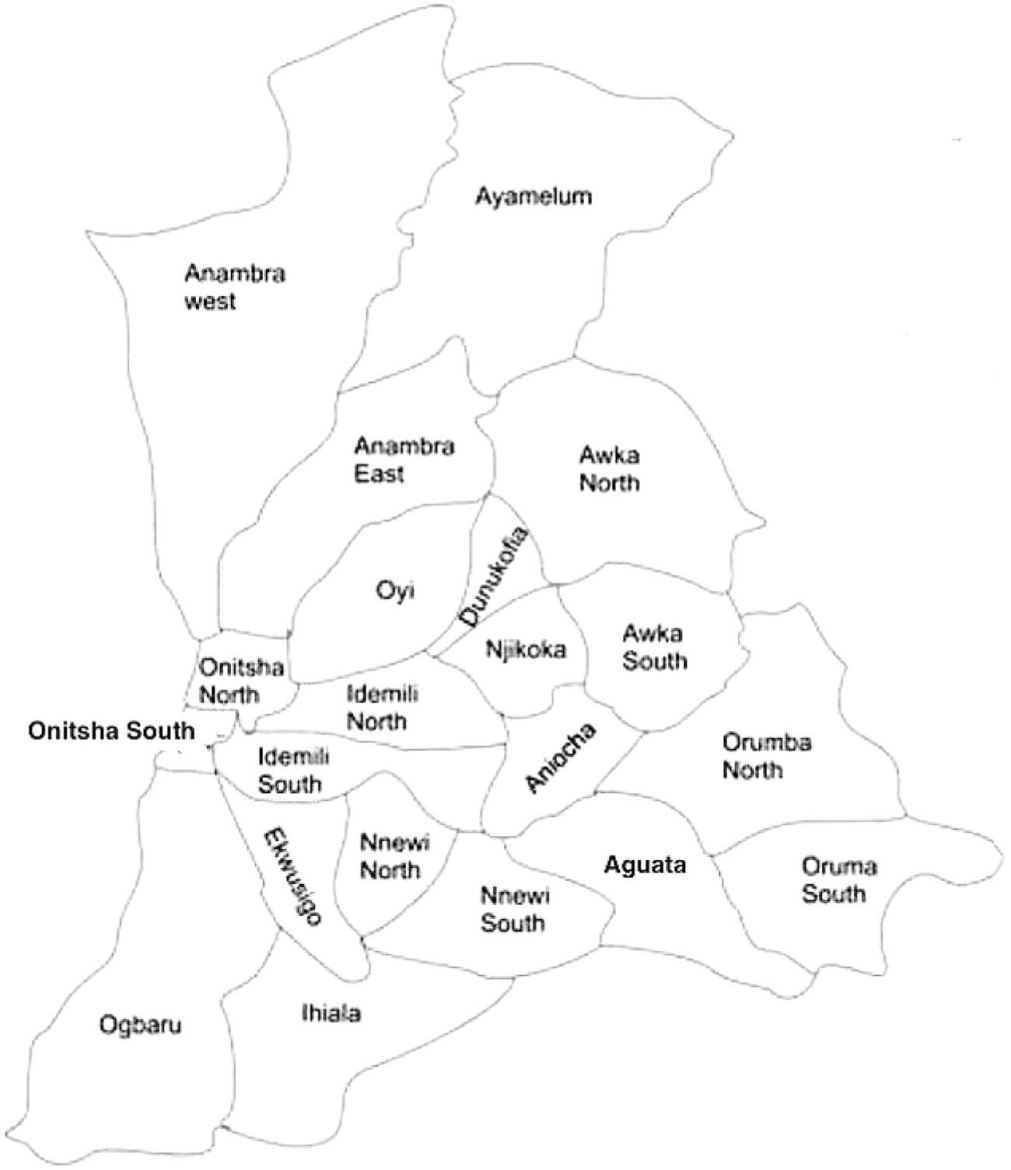
Map of Anambra State showing the 21 LGA.^13^ For this study, medical records of patients (aged 15−60) who received treatment in selected DOTS centers across Anambra State's 21 LGA between 2013 and 2017 were investigated. LGA, Local Government Areas.

A total of 1443 case files of patients (aged 15−60) who received treatment in selected DOTS centers across Anambra State's 21 LGA (Figure 1) between 2013 and 2017 were investigated. Table 1 shows the distribution and prevalence of TB and HIV/AIDS co‐infection in DOTS centers in health facilities in each of Anambra State's 21 LGA from 2013 to 2017. The highest number of cases of TB and HIV/AIDS co‐infection (23.84%) was recorded in 2014, followed by 2015 (22.80%), 2016 (20.17%), 2017 (20.03%), and 2013 (13.17%).

**Table 1:** TB and HIV/AIDS co‐infection distribution in Anambra State's 21 LGA from 2013 to 2017.

S/N	Name of LGA	Name of Facility	2013 (*n*)*	2014 (*n*)*,†	2015 (*n*)*,†	2016 (*n*)*,†	2017 (*n*)*,†	Total [n (%)]
1	Aguata	Anglican Diocesan Hospital and Maternity Igboukwu	9	21	10	10	17	67 (4.46%)
2	Anambra East	Umuleri General Hospital	7	17	15	13	13	65 (4.50%)
3	Anambra West	Oroma‐Etiti Primary Health Center	8	31	20	20	27	106 (7.35%)
4	Awka North	Isuaniocha Primary Health Center	8	27	18	12	11	76 (5.27%)
5	Awka South	Regina Caeli Hospital, Awka	11	15	31	23	23	103 (7.14%)
6	Anaocha	St. Joseph's Hospital, Adazi‐Nnukwu	16	21	22	17	18	94 (6.51%)
7	Ayamelum	Omasi‐Agu Primary Health Center	12	36	25	17	12	102 (7.07%)
8	Dunukofia	Ukpomili Health Center, Ifitedunu	13	23	16	25	8	85 (5.89%)
9	Ekwusigo	Oraifite General Hospital	15	17	12	9	10	63 (4.37%)
10	Ihiala	Our Lady of Lourdes Hospital, Ihiala	6	12	9	11	9	47 (3.26%)
11	Idemili North	Immaculate Heart Hospital, Nkpor	5	20	14	21	16	76 (5.27%)
12	Idemili South	Our Lady of Fatima Hospital and Maternity, Awka‐Etiti	4	13	21	8	8	54 (3.74%)
13	Njikoka	Enugwu Ukwu General Hospital	4	12	21	14	21	72 (4.99%)
14	Nnewi North	Nnamdi Azikiwe University Teaching Hospital, Nnewi	10	18	11	10	20	69 (4.78%)
15	Nnewi South	Ezinifite Primary Health Center	6	10	7	20	16	59 (4.09%)
16	Ogbaru	Osomalla Primary Health Center	8	9	24	15	10	66 (4.57%)
17	Onitsha North	St. Charles Borromeo Hospital, Onitsha	7	7	10	7	13	44 (3.05%)
18	Onitsha South	St. John Anglican Primary Health Care, Fegge	13	14	12	13	17	69 (4.78%)
19	Orumba North	Umunebo Primary Health Center, Ufuma	12	6	9	7	8	42 (2.91%)
20	Orumba South	Immaculate Heart Hospital, Umunze	10	8	12	11	4	45 (3.12%)
21	Oyi	Awkuzu Primary Health Center	6	7	10	8	8	39 (2.70%)
Total (*n* [%])			190 (13.17%)	344 (23.84%)	329 (22.80%)	291 (20.17%)	289 (20.03%)	1443 (100%)

*Note*: Using one‐way ANOVA, the annual prevalence rates of TB and HIV/AIDS coinfections across selected DOTS centers in Anambra State's 21 LGAs were compared from 2013 to 2017 (*) and from 2014 to 2017 (†). There was a significant difference (*p = 0.0015, *p* < 0.05) in the annual prevalence rates from 2013 to 2017; no significant difference was found between 2014 and 2017 (†*p* = 0.4506, *p* > 0.05).

Abbreviations: ANOVA, analysis of variance; HIV, human immunodeficiency virus; TB, tuberculosis.

**Figure 2:**
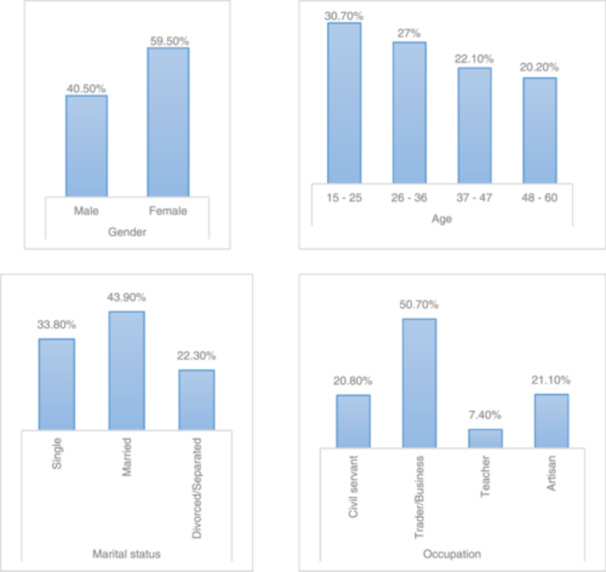
Prevalence of TB and HIV/AIDS co‐infection in Anambra State from 2013 to 2017, based on patient demographics. The study found that females made up a larger proportion (59.50%) of people coinfected with TB and HIV/AIDS compared to males (40.50%). Looking at other demographics, cases of TB and HIV/AIDS co‐infection were highest in those aged 15−25 years (30.70%), in married patients (43.90%), and in traders/business owners (50.70%). HIV, human immunodeficiency virus; TB, tuberculosis.

Figure 2 shows the prevalence of TB and HIV/AIDS co‐infection in Anambra State from 2013 to 2017, based on patient demographics. In general, 40.50% of the total study population with TB and HIV/AIDS co‐infection were males, while 59.50% were females. In terms of age, marital status, and occupation, cases of TB and HIV/AIDS co‐infection were highest in those aged 15−25 years (30.70%), in married patients (43.90%), and in traders/business owners (50.70%).

### 3.1 Annual prevalence of TB and HIV/AIDS co‐infection

Between 2013 and 2017, 1443 patients with TB and HIV/AIDS co‐infection attended the DOTS centers sampled in Anambra State. A mean prevalence rate of TB and HIV/AIDS co‐infection in the state during the 5‐year period (2013–2017) was 20.00%. The highest annual prevalence of TB and HIV/AIDS co‐infection was recorded in 2014 (23.84%). The state's prevalence of TB and HIV/AIDS co‐infection increased dramatically from 13.17% in 2013 to 23.84% in 2014, followed by a slight downward trend to 22.80% in 2015, 20.17% in 2016, and 20.03% in 2017.

The annual prevalence rate of TB and HIV/AIDS co‐infection was significantly different (*p* < 0.05) over the 5‐year period (2013‐2017), particularly due to the sharp increase from 13.17% in 2013 to 23.84% in 2014. There was no statistical difference (*p* > 0.05) in the annual prevalence rate of TB and HIV/AIDS co‐infection from 2014 to 2017, with a similar but decreasing trend of prevalence rates recorded for 2014 (23.84%), 2015 (22.80%), 2016 (20.17%), and 2017 (20.03%).

Temitayo‐Oboh et al.^4^ investigated the prevalence and factors influencing TB and HIV co‐infection among patients attending the DOTS clinic in a tertiary health center in Ogun State, Nigeria, between 2015 and 2019. Their study showed that approximately one in five TB patients was coinfected with HIV (20% prevalence), a finding consistent with our study and another study reported in Lagos, Nigeria.^14^ This prevalence was higher than rates reported in similar studies conducted in Kano (10.90%),^15^ Oyo (14.20%),^16^ and Benin City (8.40%).^6^

### 3.2 Prevalence of TB and HIV/AIDS co‐infection in relation to demographics

From 2013 to 2017, the prevalence of TB and HIV/AIDS co‐infection was higher in females (59.50%) than in males (40.50%) in Anambra State. This relative difference in the prevalence of TB and HIV/AIDS co‐infection based on the gender of patients from the same environment is not surprising, as Nwobu et al.^17^ reported a similar trend of a higher prevalence rate for females over males in a comparative study of the prevalence rates of HIV among TB patients in Irrua and Benin environs of Edo State, Nigeria.

During the 5‐year study period, patients aged 15−25 years had the highest prevalence rate of TB and HIV/AIDS co‐infection (30.70%), followed by 27% for those aged 26−36 years, 22.1% for those aged 37−47 years, and 20.20% for those aged 48−60 years. Our study found that people aged 15−25 were more likely to have TB and HIV/AIDS co‐infections. This age group represents a young population with the potential for poor sexual health and high‐risk sexual behavior, making them particularly vulnerable to TB and HIV/AIDS co‐infection.^18^ There was a general decrease in prevalence rates as patients' ages progressed from 15–25 years to 48–60 years.

Regarding patients' marital status, the prevalence rates for TB and HIV/AIDS co‐infection cases in Anambra State during the study period were 33.80% for those who are single, 43.90% for those who are married, and 22.30% for those who are divorced or separated. Increased intensity of contact, unprotected sexual activity, sharing sleeping rooms and/or one nursing the other could increase the risk of TB or HIV/AIDS for the spouse of an individual with TB and/or HIV/AIDS.^19^ The high TB and HIV/AIDS co‐infection rates among married people in this study may be due to infected married couples attending the same DOTs center, causing the records to be skewed higher for married people than for single, divorced, or separated people.

In terms of occupation, traders and business owners had the highest prevalence of TB and HIV/AIDS co‐infection cases (50.70%). This was followed by artisans (21.10%) and civil servants (20.80%). Teachers had the lowest percentage of cases (7.40%). Teachers' higher literacy levels may be linked to lower TB and HIV/AIDS prevalence, as they may have a better understanding of disease prevention and control measures, and those who are already infected with TB or HIV/AIDS may follow the treatment regimen more judiciously.

### 3.3 Implications of the findings

TB, a disease of poverty and inequality, is a leading cause of severe illness and mortality among people living with HIV. People with HIV who do not receive appropriate prevention and care are at a much higher risk of developing and dying from TB.^20^ As part of overall efforts to reduce HIV‐related morbidity and mortality in high HIV prevalence settings, the WHO developed the global framework for TB/HIV with the goal of reducing TB transmission, morbidity, and mortality (while minimizing the risk of anti‐TB drug resistance). This global framework largely focuses on Sub‐Saharan Africa.^7^

HIV has a significant impact on TB control in countries with a high TB/HIV burden. At the same time, TB is not only the leading cause of death among people with AIDS, but it is also the most common curable infectious disease among people living with HIV/AIDS. As a result, it has become clear that additional interventions are urgently needed to supplement the WHO‐recommended DOTS strategy for TB control.^7^

In Nigeria, there are DOTS centers in each of the 774 LGA where TB patients can be diagnosed and treated. Some of these centers are housed within secondary and tertiary health care facilities and are staffed by community health extension workers and/or nurses who are expected to diagnose, treat, and refer patients as needed. With the implementation of the DOTS strategy in the Nigeria, treatment success rates increased from 15% in 1995 to 86% in 2017, and case detection rates increased from 4.3% to 25.8% during the same period.^5^

Data for this study were collected from hospital records of patients who received TB and HIV/AIDS co‐infection treatments at a DOTS center selected from Anambra State's 21 LGA between 2013 and 2017. The state's prevalence of TB and HIV/AIDS co‐infection increased dramatically from 13.17% in 2013 to 23.84% in 2014, followed by a slight downward trend to 22.80% in 2015, 20.17% in 2016, and 20.03% in 2017 (Table 1).

The sharp increase in TB and HIV/AIDS co‐infection cases since 2014 is concerning, and it may reflect a failure of the state's institutional, governmental, and community TB and AIDS control systems. This could also be due to the underperformance of the state's existing DOTS centers, which may be overburdened due to a variety of factors, such as increased demand for their services as a result of a limited number of DOTS centers serving a large population, staff shortages, and so on.

According to Adebayo et al.^5^ the problems of limited access to health care services and poor quality of DOTS centers, as well as insufficient health service capacity to deliver effective TB services that will meet the needs of patients, pose a major challenge to achieving Nigeria's TB control targets. The negative consequences of the high prevalence of TB and HIV/AIDS co‐infection cases in Anambra State are obvious. Unless and until coordinated efforts are made to control the epidemic in Anambra State, including the establishment of more DOTS centers and the expansion of the capacity of existing DOTS services, it may be difficult to achieve Nigeria's and WHO's goals of reducing the national and global burden of TB and HIV/AIDS.

Despite the high prevalence rate of TB and HIV/AIDS co‐infection recorded in Anambra State during the study period, it is important to note that this study relied on records obtained from the state's DOTS centers and thus did not account for cases that were not reported or treated at these DOTS centers. These unrecorded patients may be those who, for some reason, did not visit or were unaware of the DOTS centers, or who sought alternative methods of treatment elsewhere. As a result, the prevalence of TB and HIV/AIDS in the state could be higher than reported in this paper.

According to the findings of this study, younger people, particularly those aged 15 to 36 years, as well as females, married people, and traders/business owners, appear to be the most vulnerable groups affected by TB and HIV/AIDS co‐infection, accounting for the vast majority of the disease burden in Anambra State. The implication is that unless special interventions and targeted control programs are directed to contain this growing epidemic among these groups of people, the state and even the country will lose a large proportion of its able‐bodied men and women, who constitute a percentage of its workforce or manpower resources, to the menace of TB and HIV/AIDS.

### 3.4 Recommendations

The findings of this study are expected to draw the attention of the state and local governments of Anambra State, as well as health institutions and agencies in the state and throughout Nigeria, prompting them to respond appropriately to the challenges posed by the endemic situation of TB and HIV/AIDS. This study provides further insight into the pattern of distribution of TB and HIV/AIDS co‐infection in Anambra State in terms of epidemiological variables such as age, gender, marital status, and occupation, which are associated with high rates of the diseases. This will provide the public with a clear understanding of the factors that may contribute to the spread of TB and HIV/AIDS. Empowered by this knowledge, the public can then make positive lifestyle choices and adopt positive behavioral changes toward the two endemic diseases. Massive awareness campaigns and other targeted interventions in the control and prevention of the TB and HIV/AIDS epidemics in the state should be directed at the identified vulnerable groups affected by these diseases.

## 4 CONCLUSION

Between 2013 and 2017, 1443 patients with TB and HIV/AIDS co‐infection attended the selected DOTS centers spread across Anambra State's 21 LGA. During the 5‐year period, the state had a 20.00% mean prevalence rate of TB and HIV/AIDS co‐infection, with the highest annual prevalence of 23.84% recorded in 2014. The most vulnerable groups affected by TB and HIV/AIDS co‐infection appear to be young people aged 15−25, females, married people, and traders/business owners, accounting for the vast majority of disease burden in Anambra State. To address the state's high prevalence of TB and HIV/AIDS co‐infection, more intervention and control programs must be implemented, and those that are already in place must be made much more effective.

## AUTHOR CONTRIBUTIONS

**Monique Okeke**: Data curation; formal analysis; investigation; methodology; project administration; resources; writing—original draft; writing—review and editing. **Peter M. Eze**: Data curation; formal analysis; software; writing—original draft; writing—review and editing. **Adaeze E. Chukwudebelu**: Formal analysis; writing—review and editing. **Chidiebere J. Nwankwo**: Formal analysis; writing—review and editing. **Nchekwube K. Eze**: Formal analysis; writing—review and editing. **Uchenna U. Okafor**: Formal analysis; writing—review and editing. **Isaiah C. Abonyi**: Formal analysis; writing—review and editing. **Eric E. Okereke**: Formal analysis; writing—review and editing. **Kalu O. Obasi**: Formal analysis; writing—review and editing. **Okorie A. Ede**: Formal analysis; writing—review and editing. **Chika P. Ejikeugwu**: Formal analysis; writing—review and editing. **Cajetan I. Ilo**: Data curation; formal analysis; methodology; supervision; writing—review and editing. **Jerome O. Okafor**: Conceptualization; methodology; supervision; writing—review and editing.

## CONFLICT OF INTEREST STATEMENT

The authors declare no conflict of interest.

## ETHICS STATEMENT

Ethical approval for this study was provided by the Ethical Board of Anambra State Ministry of Health, Nigeria (Approval number: MH/PRS/986/47).

## TRANSPARENCY STATEMENT

The lead author Monique Okeke affirms that this manuscript is an honest, accurate, and transparent account of the study being reported; that no important aspects of the study have been omitted; and that any discrepancies from the study as planned (and, if relevant, registered) have been explained.

## Data Availability

The data that support the findings of this study are available from the corresponding author upon reasonable request.
